# Contemporary Issues in Postmastectomy Radiotherapy: A Brief Review

**DOI:** 10.3390/jcm13247545

**Published:** 2024-12-11

**Authors:** Caroline A. Grace, Michael J. McKay

**Affiliations:** 1 Northwest Regional Hospital, Rural Clinical School, The University of Tasmania, Burnie, TAS 7320, Australia; 2 Northern Cancer Service, North West Cancer Centre, Burnie, TAS 7320, Australia

**Keywords:** mastectomy, radiotherapy, breast cancer, breast reconstruction, hypofractionation

## Abstract

Breast cancer is the one of the most common cancers and causes a significant disease burden. Currently, postmastectomy radiotherapy (PMRT) is indicated for breast cancer patients with higher risk of recurrence, such as those with positive surgical margins or high-risk breast cancer (T3 with positive lymph nodes, ≥4 positive lymph nodes or T4 disease). Whether PMRT should be used in intermediate-risk breast cancer (T3 with no positive lymph nodes or T1-2 with 1-3 positive lymph nodes) is contentious. Rates of breast reconstruction postmastectomy are increasing in countries like Australia, and PMRT usage in such settings is another area of active research. Ongoing trials are also assessing the safety and efficacy of hypofractionated PMRT, a clinical scenario now widely accepted for early-stage breast cancer. This brief review is unique in that it aims to examine three current and controversial aspects of the PMRT field (PMRT in intermediate-risk breast cancer, PMRT in conjunction with breast reconstruction and its hypofractionation). To achieve this aim, we discuss available and emerging literature and guidelines to offer insights important to the PMRT field. Current literature suggests that PMRT could play a role in improving the overall survival rate and in reducing locoregional recurrence in intermediate-risk breast cancer. In terms of recommending a timing or type of breast reconstruction best suited to the setting of PMRT, we found that individual patient preferences and circumstances need to be considered alongside a multidisciplinary approach. Research into PMRT hypofractionation safety and efficacy is ongoing and its place remains to be elucidated.

## 1. Introduction

**Figure 1:**
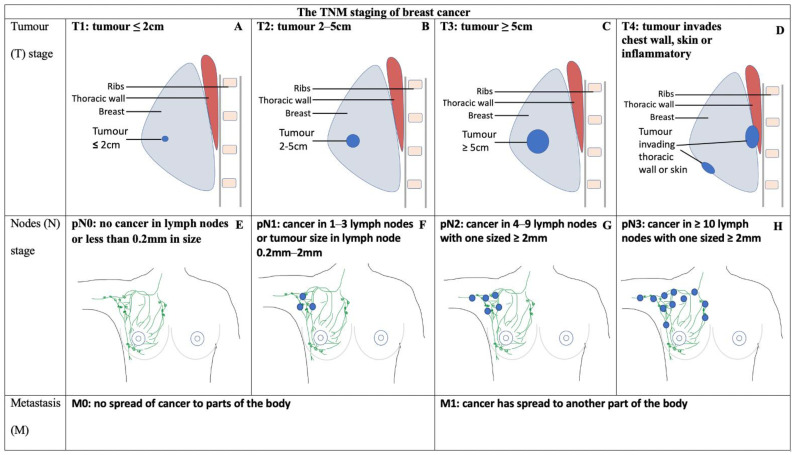
A diagrammatic representation of the TNM staging of breast cancer. Rows from top to bottom represent tumour, nodal and metastatic classifications of breast cancer. Tumours and affected lymph nodes are represented by blue-shaded circles for the tumour and nodal rows, respectively. Intermediate-risk breast cancer is classified by St Gallen [6] as E+B (pT2N0), E+C (pT3N0), A+F (pT1N1), B+F (pT2N1) or C+F (pT3N1). Diagrams are original, created by C.A.G. using PowerPoint and adapted from Cancer Research UK [7].

**Figure 2:**
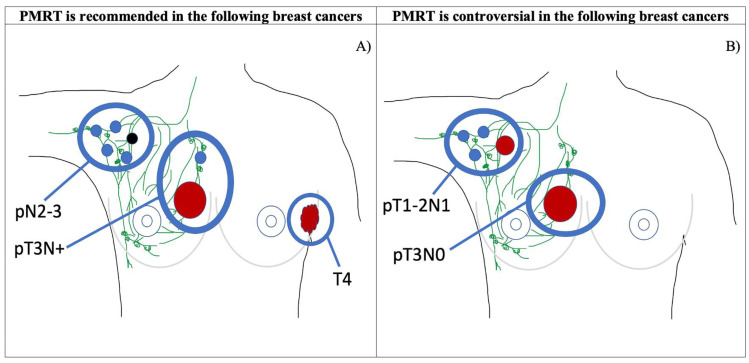
A diagrammatic summary of the current and controversial indications of PMRT based on current PMRT guidelines from NCCN, ASCO and EviQ. (**A**) Apart from positive margins, PMRT is currently recommended for pN2-3 (≥4 nodes affected), pT3N+ (T3 with positive node(s)) and T4. (**B**) PMRT’s use in pT1-2N1 (T1-2 with <4 nodes affected) and T3N0 (T3 with no nodes affected) is controversial. Small blue-shaded circles represent affected lymph nodes (black circle represents pN3), red shapes representing breast cancer. Diagrams were created by C.A.G. using PowerPoint and adapted from Cancer Research UK [7].

Breast cancer is the one of the most common cancers amongst women and causes a significant disease burden [1]. Treatment modalities for breast cancer vary from breast-conserving surgery (BCS) or mastectomy to chemotherapy, endocrine therapy, targeted therapy or immunotherapy, depending on patient preference and the cancer histopathology and staging [1,2,3]. A mastectomy may be performed as per patient preference or when BCS is contraindicated or unsuccessful [1]. Local guidelines on postmastectomy radiotherapy (PMRT) are provided by the Australian organisation EviQ [2], whilst international guidelines are provided by the National Comprehensive Cancer Network (NCCN) [3] and the American Society of Clinical Oncology (ASCO) [4,5]. The classification and staging of breast cancers, as well as the indications for PMRT are summarised in Figure 1, Figure 2 and Table 1.

Currently, PMRT is recommended in invasive breast cancer where there are positive surgical margins and in high-risk breast cancer (T3 with at least 1 positive lymph node, ≥4 positive lymph nodes (pN2-3) or T4) [2,3,4,5]. In these cases, PMRT has been shown to confer a significant benefit in reducing both the locoregional recurrence risk and mortality of breast cancer [2,3,4,5]. It is important to note that there is no doubt that PMRT is recommended in all high-risk breast cancer except for the case of HER 2-positive pN1 (1-3 nodes affected), for which PMRT is not explicitly recommended [2,3,4,5]. Interestingly, pN1 tumours could be classed as intermediate or high-risk, depending on HER 2 receptor status [6]. For this review, we have grouped pN1 with intermediate-risk breast cancer, as there are limited papers examining PMRT’s effect on receptor subtypes within pN1 breast cancer. Whether PMRT should be recommended for intermediate-risk breast cancer (T3 with no positive lymph nodes or T1-2 with 1-3 positive lymph nodes (pN1)) is controversial [2,3,4,5]. This brief review examines three current and controversial aspects of the PMRT field including PMRT in intermediate-risk breast cancer, PMRT in conjunction with breast reconstruction and its hypofractionation.

## 2. The Use of PMRT in Intermediate-Risk Breast Cancer (T3N0 or T1-2N1)

The use of PMRT in patients with intermediate-risk breast cancer is topical and controversial. The various studies examining the issue are listed in Table 2.

In 2023, a St Gallen International Consensus Conference for the Primary Therapy of Individuals with Early Breast Cancer was held. At this conference, panellists recommended PMRT in T2 disease depending on the amount of lymph nodes affected and tumour receptor subtype. In the setting of T3N0 disease, tumour receptor subtype also influenced recommendations for PMRT, with fewer expert panellists recommending PMRT for ER-positive breast cancer (49%) than for HER 2-positive or triple-negative subtypes (>80%) [8].

T3N0M0 breast cancer comprises a relatively small proportion of new breast cancer diagnoses [9,10]. One study analysed the rates of recurrence for 313 patients with T3N0M0 cancer who were managed with mastectomy but without PMRT. These patients were a part of five various trials overseen by the National Surgical Adjuvant Breast and Bowel Project. Of the included patients, 46% had a 5cm-sized breast tumour, whilst 54% had a tumour larger than 5 cm. The reported 10-year rate of isolated locoregional failure as an initial incident was 7.1%, locoregional failure with/without distant failure as an initial incident was 10.0% and isolated distant failure as an initial incident was 23.6%. Multivariate analysis found no independent prognostic factors for distant or locoregional failure. The authors suggested that since patients who went without PMRT had low locoregional recurrence, PMRT does not have a place in the routine management of T3N0M0 breast cancer [11].

However, several other large retrospective studies investigating PMRT’s utility in T3N0M0 breast cancer reached different conclusions. Many of these studies analysed the large databases of the “Surveillance, Epidemiology and End Results Program (SEER)” or the “National Cancer Data Base (NCDB)” to reveal associations between PMRT and improved locoregional control and overall survival (OS) in T3N0M0 cancers. Johnson et al. performed a retrospective analysis using the SEER database, which included 2525 patients, of whom 1063 received PMRT. PMRT was associated with a statistically significant improvement in OS. At 8 years, OS was 76.5% for PMRT in comparison to 61.8% for the cohort who did not undertake PMRT [12]. Using the NCDB, Francis et al. analysed the 5-year OS rate in 4291 patients with T3N0M0 breast cancer. All patients underwent a mastectomy, and 47% of these then received PMRT. The OS in patients managed with PMRT was 83.7%, compared to the OS rate of 79.8% in the cohort without PMRT, a statistically significant difference. A significant benefit in OS was maintained at 10 years with an overall OS rate of 67.4% for patients who received PMRT in comparison to 59.2% for those who did not [13]. An OS benefit associated with PMRT was also reported in an analysis of patients identified in the NCDB performed by Cassidy et al. This study involved 3437 patients with pT3N0M0 breast cancer who were managed with a mastectomy. Of the 3437 patients, 1051 received PMRT isolated to the chest wall whilst 550 received chest wall and regional nodal radiation. The group who was managed with PMRT had a statistically significant improvement in their 5-year OS rate (86.3%) compared to the group who did not receive PMRT (66.4%). Importantly, the improvement in OS associated with PMRT happened regardless of whether patients underwent systemic treatments like hormone treatment or chemotherapy [14].

Various patient characteristics and other treatment factors may modulate the advantage of using PMRT in T3N0M0 breast cancer. One retrospective study used the SEER database to examine OS rates in patients with T3N0M0 breast cancer according to age. The analysis included 4840 patients diagnosed with T3N0M0 breast cancer between 2000 and 2015, of whom 2243 patients received PMRT. A significantly higher OS rate was identified for older patients (>55 years) who underwent PMRT, compared to those who did not. However, for younger patients (≤55 years), PMRT was not associated with any changes in OS [15]. The results of a matched-cohort analysis using data from the NCDB indicated that the improvement in OS associated with PMRT may be restricted to cohorts not undergoing adjuvant chemotherapy. This study involved 13,901 patients with T3N0 breast cancer. The 7-year OS was significantly higher for the cohort who underwent PMRT compared to no PMRT (74% vs. 65%). However, PMRT did not significantly influence OS rates for patients receiving additional treatment with adjuvant or neo-adjuvant chemotherapy [16]. This is in contrast to a study conducted by Overgaard et al., who analysed 1375 patients in the Danish Breast Cancer Cooperative Group 82b and 82c randomised controlled trials [17]. In total, 689 received tamoxifen (30 mg daily for 1 year) alone whilst 686 received tamoxifen plus PMRT. The 10-year LRR was 8% in the PMRT + tamoxifen group and 35% for the tamoxifen-only group, whilst the 10-year OS was 45% in the PMRT + tamoxifen group and 36% for the tamoxifen-only group (*p* = 0.03). These findings reflect Cassidy et al.’s analysis of the NCDB, which revealed an improved OS irrespective of systemic therapy [14]. Therefore, factors such as age or adjuvant chemotherapy may modulate PMRT’s efficacy in intermediate-risk breast cancer, although further research is needed in this area.

Limited prospective evidence supporting PMRT’s use in T3N0M0 breast cancer exists. The Danish Breast Cancer Cooperative Group 82b and 82c randomised controlled trials reported a significant positive association between PMRT and reduced rates of locoregional recurrence and a positive effect on OS among patients with stage 2 and 3 breast cancer. However, these studies comprised a relatively small number of patients with T3 or N0 breast cancer, and the analyses were unplanned (post-hoc) [17,18]. In summary, the NCCN guidelines encourage PMRT to be considered for T3N0 breast cancer, while the ASCO guidelines do not make specific recommendations on PMRT for this patient group [3,4,5].

PMRT in the breast cancer setting of T1-2 tumours with 1-3 positive lymph nodes (pN1) is also contentious. A meta-analysis of 8135 patients across 22 trials suggested that PMRT should be recommended in pN1 tumours. All patients were managed with a mastectomy and were grouped into whether they had received PMRT or not. Results showed that PMRT had a statistically significant association with reduced breast cancer mortality amongst 1314 patients who had pN1 [19]. Another 2018 post-hoc study made on randomised data analysed the outcomes of 684 patients with T1-2N1 breast cancer who had been included in the Breast International Group 02-98 trial. In this analysis, locoregional recurrence rates were significantly lower at 10 years for the cohort who underwent PMRT compared to patients who had not. Interestingly PMRT was not associated with any significant changes in OS [20].

The NCCN guidelines recommend considering PMRT in patients with 1-3 positive lymph nodes on axillary dissection. The revised ASCO guidelines on PMRT were released in 2016 addressing the use of PMRT for patients with T1-2 breast cancer and 1-3 positive axillary lymph nodes [5]. This update recommended consideration of factors that may lower the rate of locoregional recurrence (such as T1 tumour size or only a single positive lymph node), lessen the advantage of reduced breast cancer-specific mortality (such as limited life expectancy) and increase the risks of radiotherapy complications through the presence of co-existing medical conditions. These guidelines also recommend that the decision to receive PMRT should be made in discussion with a multidisciplinary team and the patient.

Results from the SUPREMO randomised controlled trial may elucidate the benefit of PMRT in patients in this group. This study included 1688 breast cancer patients who underwent a mastectomy. Patients were randomised to either PMRT or no radiotherapy between 2006 and 2013. Patients with pT1-2N1 breast cancer as well as pT2N0 disease if also grade 3 with lymphovascular invasion were included. Patients with T3N0 breast cancer were eligible, but made up a small proportion of the patient cohort (<1%). The primary outcome for this study is 10-year overall survival. A quality-of-life analysis for SUPREMO at a 2-year follow-up reported a small but significant increase in chest wall symptoms (skin problems, pain oversensitivity and swelling) in the PMRT group, but no difference in other quality-of-life indices, including shoulder symptoms, body image and overall quality of life [21]. Survival outcomes from this study are yet to be published.

Recently, NCCN and ASCO guidelines have considered the use of recurrence probability tests such as the Oncotype DX recurrence score (RS) in determining whether PMRT should be recommended in intermediate-risk breast cancer [3,4,5]. Oncotype DX is a genomic test performed on an individual’s breast tumour. It is a 21-gene RS which has been used to predict the response to chemotherapy, particularly in ER-positive early breast cancer [22,23]. There are limited studies examining PMRT and RS usage. However, Larson et al.’s study involving 150 women with pT1N0 breast cancer revealed that RS may have a beneficial role in guiding intra-operative radiotherapy use for breast cancer [23]. Admittedly, this study looked at low-risk breast cancer, and the radiotherapy was delivered intra-operatively not post-operatively. Another study used the National Cancer Database and applied this RS to the PMRT situation, asking whether the RS could predict overall survival after PMRT. The 5-year OS in 8907 patients with pN1 breast cancer and an RS < 25 was examined, and the lack of PMRT had no effect on overall survival. The authors concluded that the RS may have efficacy in intermediate breast cancer risk situations where PMRT is being considered. Furthermore, they suggested that, based on their data, de-escalation of PMRT may be possible. However, they did not suggest a de-escalation strategy nor report on locoregional control, a key post-PMRT outcome [24]. This implies that the controversies of PMRT usage in intermediate-risk breast cancer may not be resolved by RS. Further research into RS and PMRT is needed to confirm the role of RS in informing PMRT’s use in intermediate-risk breast cancer.

Multifocality (more than one tumour in a single breast quadrant) and multicentricity (more than one tumour in more than one breast quadrant) have been suggested to be risk factors for local PMRT recurrence. This was examined in a post-hoc analysis of the EORTC 22922 and MA20 clinical trials by Mamtani et al. (2017), who evaluated T1-T2N0 breast cancer cases with at least one high-risk feature (including multifocality) [25]. Multifocality, in addition to other high-risk features, did not increase locoregional recurrence risk: the authors suggested that these features were not themselves indicators for the need for PMRT. This was confirmed in another study, in that multifocality was also not found to be an independent risk factor for locoregional breast cancer recurrence after PMRT [26].

As aforementioned, the 2023 St Gallen Conference panellists recommended considering HER 2 receptor positivity as a factor which may increase the benefit of PMRT in intermediate-risk breast cancer [8]. Additionally, HER 2 receptivity influences whether a pN1 breast cancer is classified as an intermediate or high-risk breast cancer (see Table 1) [6]. Despite this distinction in breast cancer classification, current guidelines do not explicitly recommend PMRT in pN1 HER 2 receptor-positive breast cancer [2,3,4,5]. Since PMRT is designed to reduce recurrence rates and high-risk breast cancers have higher recurrence rates, we explored this specific receptor in the setting of PMRT [2].

One study analysed the 5-year locoregional recurrence rate in 5442 patients spread over 11 hospitals. Patients had pT1-2N1 breast cancer and were divided into HER 2-negative and HER 2 over-expressing cohorts. With and without PMRT, locoregional recurrence rates in the HER 2-negative cohort were 1.9% and 6.5%, respectively (*p* < 0.001), whilst rates in the HER 2 over-expressing cohort were 10.2% and 15.5%, respectively (*p* = 0.236). Interestingly, in triple-negative pT1-2N1 breast cancer, locoregional recurrence for the PMRT cohort was 4.6%, and in the unirradiated group, it was 15.9% (*p* = 0.002) [27]. This suggests that PMRT has a role in reducing locoregional recurrence in pT1-2N1 HER 2 receptor-negative or triple-negative breast cancer but not in HER 2 receptor over-expressing breast cancer.

Another study analysed 1000 patients who were a part of the Danish Breast Cancer Cooperative Group (DBCG) protocol 82 trials b and c. Although all patients had high-risk breast cancer, it was found that PMRT was not associated with an improved OS for HER receptor positivity; however, in triple-negative breast cancers, it conferred an improvement which was statistically significant [28]. Again, there was another study using the SEER database to analyse the outcomes of 1887 patients who received PMRT. The majority of patients were pN1- (12.8%) and HER 2-negative (92.7%), with the median follow-up time being 28 months. Patients had a higher OS when they received PMRT and were HER 2-negative, whereas PMRT did not confer a statistically significant benefit for OS in patients with HER 2-positive breast cancer [29]. It is counterintuitive that PMRT would confer little benefit to HER 2 receptor-positive breast cancer. Research conducted in 1999 revealed that HER 2 receptor-positive breast cancer cells are actually radiation-resistant, suggesting that adjuvant radiation treatment may be of benefit in this setting [30]. More studies should be conducted to determine if receptor subtypes may inform PMRT use in intermediate-risk breast cancer.

**Table 2:** A tabulated summary of the content in Section 2 “The use of PMRT in intermediate-risk breast cancer (T3N0 or T1-2N1)”. Each row is a different resource which examines PMRT’s role in either pT3N0 or pT1-2N1 breast cancers. Each column of the table includes details about the resource, including author names/reference, databases, patient numbers, locoregional recurrence, overall survival results and other notes.

Breast Cancer Stage	Author/Database	Sample	Locoregional Recurrence (LRR)	Overall Survival (OS)	Notes
T3 N0	Almahariq MF et al. using NCDB (2020) [16]	13901 patients	Not stated	Seven-year OS was 74% in PMRT group and 65% for the no-PMRT group (*p* < 0.001). However, for patients who were also treated with adjuvant or neo-adjuvant chemotherapy, there were no significant differences in OS for those managed with PMRT.	Improvement in OS associated with PMRT may be restricted to patients who do not undergo adjuvant chemotherapy.
	Cassidy et al. using the NCDB (2017) [14]	3437 patients, 47.8% received PMRT	Not stated	Five-year OS was 86.3% in PMRT group and 66.4% for the no-PMRT group (*p* < 0.01). Improved OS in PMRT group independent of whether patients underwent systemic treatment.	Authors recommend PMRT.
	Kwong et al. as a part of Early Breast Cancer Trialists’ Collaborative Group (2014) [19]	8135 patients who had mastectomy and axillary surgery as a part of a meta-analysis of 22 trials; 700 patients were N0	PMRT had no significant effect on 10-year LRR (2-tailed *p* value > 0.1).	PMRT had no significant effect on 20-year breast mortality (2-tailed *p* value > 0.1).	Authors do not recommend PMRT.
	Francis et al. using the National Cancer Data Base (NCDB) (2017) [13]	4291 patients, 47% received PMRT	Not stated	Five-year OS was 83.7% in PMRT group and 79.8% for the no-PMRT group (*p* < 0.001). Ten-year OS was 67.4% in PMRT group and 59.2% for the no-PMRT group (*p* < 0.001).	Authors recommend PMRT to improve OS.
	He et al. using the SEER database (2021) [15]	4840 patients, 2243 patients received PMRT	Not stated	OS rate was significantly higher in patients older than 55 years who were treated with PMRT (*p* < 0.001) For patients 55 years or younger, there was no significant difference in OS for PMRT.	Authors suggest PMRT’s benefit is related to age.
	Johnson et al. using the Surveillance, Epidemiology and End Results Program (SEER) database (2014) [12]	2525 patients, 1063 received PMRT	Not stated	Eight-year OS was 76.5% in PMRT group and 61.8% for the no-PMRT group (*p* < 0.01).	Authors encourage strong consideration of PMRT.
	Neilsen et al. with the Danish Breast Cancer Cooperative Group 82b and 82c randomised controlled trials (2006) [18]	3083 patients	Eighteen-year LRR was 14% in PMRT group and 49% for the no-PMRT group (*p* < 0.001).	Not stated	Authors recommend PMRT to reduce LRR.
	Overgaard et al. with the Danish Breast Cancer Cooperative Group 82b and 82c randomised controlled trials (1999) [17]	1375 patients, 689 received tamoxifen (30 mg daily for 1 year) alone, whilst 686 received tamoxifen plus PMRT to the chest wall and regional lymph nodes	Ten-year LRR was 8% in PMRT + tamoxifen group and 35% for the tamoxifen-only group (*p* < 0.001).	Ten-year OS was 45% in PMRT + tamoxifen group and 36% for the tamoxifen-only group (*p* = 0.03).	Authors recommend PMRT with tamoxifen to reduce LRR and increase OS.
	St Gallen International Consensus Conference for the Primary Therapy of Individuals with Early Breast Cancer (2023) [8]	N/a	Not stated	Not stated	Panellists recommend PMRT for HER 2+ or triple-negative subtypes (>80%) compared to ER-positive breast cancer (49%).
	Taghian et al. using the National Surgical Adjuvant Breast and Bowel Project trials B-13, B-14, B-19, B-20, and B-23 (2006) [11]	313 patients over 5 different trials who did not receive PMRT	Ten-year rate for isolated LRR is 7% in tumours 5 cm in size and 7.2% for tumours larger than 5 cm (*p* = 0.9).	Not stated	Authors do not recommend PMRT due to low rates of LRR postmastectomy alone
T1-2 N1	Kwong et al. as a part of Early Breast Cancer Trialists’ Collaborative Group (2014) [19]	8135 patients who had mastectomy and axillary surgery as a part of a meta-analysis of 22 trials; 1314 were T2N1	PMRT reduced 10-year LRR (2-tailed *p* value < 0.00001). PMRT reduced LRR in the presence of systemic therapy (2-tailed *p* value < 0.00001).	PMRT reduced 20-year breast cancer mortality (RR 0.80, 95% CI 0.67–0.95, 2-tailed *p* value = 0.01) PMRT reduced 20-year breast cancer mortality (RR 0.78, 95% CI 0.64–0.94, 2-tailed *p* value = 0.01) in the presence of systemic therapy.	Authors recommend PMRT, even in the presence of systemic therapy.
	Velikova et al. as a part of the SUPREMO randomised controlled trial (2018) [21]	1688 breast cancer patients; 1% had T3N0 but the rest were T1-2N1	Not stated	Results for 10-year OS are still to come.	Quality-of-life analysis for SUPREMO at 2-year follow-up reported a small but significant increase in chest wall symptoms in the PMRT group.
	Guo X et al. using data collected from 11 Chinese Hospitals (2022) [27]	5442 patients.	Five-year LRR was 1.9% in the PMRT group and 6.5% for the no-PMRT group (*p* < 0.001) for the HER 2-negative cohort. Five-year LRR was 10.2% in the PMRT group and 15.5% for the no-PMRT group (*p* = 0.236) for the HER 2 overexpressing cohort. Five-year LRR was 4.6% in the PMRT group and 15.9% for the no-PMRT group (*p* = 0.002) for the triple-negative cohort.	Not stated	Authors suggest PMRT has a role in reducing LRR in pT1-2N1 HER 2 receptor-negative or triple-negative breast cancer but not in HER 2 receptor over-expressing breast cancer.
	Halfteck et al., using the NCDB (2023) [24]	8907 patients with pN1 breast cancer and an RS < 25; 36% received adjuvant PMRT	Not stated	Across all cohorts, the 5-year OS was 97.5% in PMRT group and 96.8% for the no-PMRT group (*p* = 0.063).	Suggests that the RS may have efficacy in intermediate breast cancer risk situations where PMRT is being considered. De-escalation of PMRT may be possible.
	Hu J et al., using the SEER database (2022) [29]	1887 patients who received PMRT; majority of patients were pN1 (12.8%) and HER 2-negative (92.7%)	Not stated	PMRT improved OS in HER 2 receptor-negative breast cancer (*p* = 0.017). PMRT did not improve OS in HER 2 receptor-positive breast cancer (*p* = 0.298).	Suggests that PMRT has a role in improving OS in pT1-2N1 HER 2 receptor-negative breast cancer but not in HER 2 receptor-positive breast cancer
	Zeidan et al. using the Breast International Group 02-98 trial (2018) [20]	684 patients, 337 (49%) received PMRT	Ten-year LRR was 2.5% in the PMRT group and 6.5% for the no-PMRT group (*p* = 0.005).	Ten-year OS was 81.7% in PMRT group and 78.3% for the no-PMRT group (*p* = 0.47).	Authors recommend PMRT to reduce LRR.

## 3. The Use of PMRT in Breast Reconstruction

Patients may choose to have a breast reconstruction post-mastectomy. Indeed, the number of breast reconstructions post-mastectomy has increased in countries like Australia over recent decades [31,32]. There are two main techniques that may be employed: autologous reconstructions, which use the patient’s own skin, fat and muscle, and prosthetic implants, which use a foreign material. In addition, the reconstruction can be performed immediately (during the operation for the mastectomy) or in a delayed fashion (after the mastectomy surgery is completed). An immediate breast reconstruction has been shown to reduce the number of operations required and may avoid the psychological impact of time spent without a breast post-mastectomy [33]. A delayed reconstruction is performed during a separate surgery following mastectomy. This results in a shorter initial mastectomy surgery and associated recovery, while providing time for the patient to consider options for reconstruction. Furthermore, if PMRT is required, the flap or implant is not exposed to potential adverse effects of radiotherapy [34]. A third option, immediate-delayed reconstruction, involves placing a tissue expander prior to a separate reconstructive surgery, which may be useful as a temporising breast mound in cases where the need for PMRT may not be confirmed until the histology of the mastectomy is available [35].

Implant-based breast reconstruction is a more popular method than autologous reconstruction, with benefits including lack of morbidity associated with the donor site, a shorter procedure and a shorter patient recovery time [36]. However, multiple studies, including one recent systematic review, have reported greater satisfaction with aesthetic outcomes following autologous reconstruction [37]. Furthermore, implant reconstructions are associated with elevated complication risks in the setting of PMRT. The Mastectomy Reconstruction Outcomes Consortium (MROC) study was a large multicentre cohort study performed in the USA [38]. The study assessed outcomes for patients who had breast reconstruction post-mastectomy and included 2247 patients, of which 622 received PMRT. Patients who received autologous reconstruction were at significantly lesser risk of developing complications at two years post-operation in comparison to those who received implant-based reconstruction.

The timing of PMRT in the setting of a breast reconstruction is not always clear. Despite its anti-cancer role, radiation therapy can negatively impact reconstruction results, for instance causing fibrosis, fat necrosis, shrinkage and capsular contracture of tissue around an implant [39]. For patients who receive implant-based reconstruction, rates of severe capsular contracture reported in one systematic review including 26 studies was 25% for radiotherapy prior to reconstruction and 32% for radiotherapy received following reconstruction, a difference which was not statistically significant [40]. A meta-analysis of six articles examined the outcomes of 1234 reconstructed breasts, 391 of which were exposed to PMRT. Results demonstrated that PMRT increased susceptibility to deep wound infection (*p* = 0.001), loss of implant (*p* = 0.009) and capsular contracture (*p* = 0.001) [41]. Another study suggested that having previous PMRT can impact breast reconstruction surgery. The study analysed 199 patients and revealed that PMRT increased rates of surgical complications (40% in the PMRT group vs. 20.2% in the non-irradiated group) and wound dehiscence (11% in the PMRT group vs. 3% in the non-irradiated group) [42]. Thus, PMRT can elevate adverse events in breast reconstruction.

Conversely, breast reconstruction can affect PMRT efficacy and delivery. Motwani et al. analysed 110 patients who had a mastectomy with immediate reconstruction and matched them with patients of the same stage who had a mastectomy but no reconstruction. A scoring system based on evaluating the delivery and safety of PMRT planning was developed and used for each patient. Results revealed that PMRT planning was compromised in 52% of patients with breast reconstructions as compared to 7% in patients without a reconstruction. The authors suggest that this is due to increased complexity in tissue integrity in a reconstructed breast which decrease radiation to affected tissue and increase incidental radiation exposure to the heart and lungs. Unfortunately, this study did not hypothesise or examine how such plans would affect locoregional recurrence or OS amongst cohorts [43].

To avoid potential radiotherapy-associated adverse effects for autologous tissue reconstruction, delayed reconstruction or a delayed reconstruction with a tissue expander has previously been preferred for patients who require PMRT [44]. However, an analysis of the MROC study regarding the outcomes of 175 patients who received PMRT who underwent either an immediate or delayed free-abdominal-based autologous breast reconstruction reported no significant difference in complication rate, aesthetic outcome or patient satisfaction between the two groups [45].

The use of hypofractionated PMRT in breast reconstruction is also a current topic of investigation. The FABREC study is a prospective randomised trial assessing self-reported outcomes among patients with stage 0–3 breast cancer managed with mastectomy and immediate breast implant or tissue expander who received either conventionally fractionated or hypofractionated radiotherapy. Conventionally fractionated PMRT involved 50 Gy in 25 fractions to the chest wall, while hypofractionated PMRT consisted of 42.56 Gy in 16 fractions. At a median follow-up of 31.8 months, there were no significant differences in physical well-being or rates of chest wall toxicities between the different PMRT fractionations; longer-term results are awaited [46]. A 5-year analysis of a smaller prospective trial assessed rates of severe radiotherapy-associated toxicities for 67 patients with stage 2-3A breast cancer who received hypofractionated PMRT with 36.63 Gy in 11 fractions with an optional four fractions of 3.33 Gy mastectomy scar boost. No acute or late grade 3 or 4 non-reconstruction-related adversities were reported in this analysis. Forty patients included in this study underwent breast reconstruction prior to PMRT, and three had breast reconstruction post-radiotherapy. Among these 43 patients, grade 3 or 4 radiotherapy-related reconstruction complications were reported at a rate of 35% [47]. Following on from this study, the RT-CHARM randomised controlled trial is currently investigating outcomes for patients receiving conventionally fractionated and hypofractionated PMRT before or after breast reconstruction [48].

Ultimately, in addition to the need for PMRT, multiple factors will influence the approach to breast reconstruction following mastectomy. The most appropriate type of reconstruction and timing in relation to radiotherapy will vary according to each patient’s individual circumstances. The decision should be made by the patient in consultation with a multidisciplinary team [44].

## 4. Hypofractionation of PMRT

Hypofractionated radiotherapy involves the delivery of more than 2 Gy per fraction [49]. The various studies examining the issue are listed in Table 3. Evidence supporting hypofractionation for PMRT is emerging. Outcomes for conventionally fractionated (50 Gy in 25 fractions) treatment were evaluated against hypofractionated adjuvant radiotherapy regimens in the START A and START B trials, which included patients with pT1-3, pN0-1 and M0 breast cancers [49]. Results from both the START A and START B trials demonstrated no significant difference in rates of local–regional relapse between conventionally fractionated and hypofractionated radiotherapy. However, most patients enrolled in these trials received breast-conserving surgery, and only a low number of patients had a mastectomy (336 patients in the START A trial (15%) and 177 patients (8%) in the START B trial).

Survival outcomes and rates of toxicity for patients treated with hypofractionated PMRT have been analysed in several retrospective studies (Table 3). One analysis of 133 patients managed with hypofractionated PMRT, 40 Gy in 16 fractions, reported a 5-year local recurrence-free rate of 97.6%, a 5-year OS rate of 74.6% and a 5-year breast cancer survival rate of 77.7%. The reported rates of severe acute toxicities were low, with only one incidence of grade 3 chest wall pain [50]. Another single-centre retrospective study compared outcomes for 334 patients who received hypofractionated PMRT to 128 patients who underwent conventionally fractionated PMRT. At a median follow-up of 65.7 months, there was no significant difference in 5-year locoregional recurrence-free survival, 5-year disease-free survival, 5-year OS or toxicity rates [51].

The efficacy of hypofractionated PMRT in high-risk breast cancer has been prospectively assessed in the RT CHARM trial: a phase III randomised controlled study where 820 patients with T3-4 breast cancer or pN2-3 were randomised to either 50 Gy in 25 fractions or 43.5 Gy in 15 fractions PMRT. This trial suggested no differences in 5-year locoregional recurrence between hypofractionation (8.3%) and conventional fractionation (8.1%) for PMRT. There were also no significant differences in acute or late toxicities other than a higher rate of acute skin reaction in the conventional fractionation group. It is important to note that patients who underwent breast reconstruction were excluded from this trial [52].

**Table 3:** A tabulated summary of the content in Section 4 “Hypofractionation of PMRT”. Each row is a different resource which examines conventional or hypofractionated PMRT. Each column of the table includes details about the resource, including author names/reference, databases, patient numbers, locoregional recurrence, overall survival, toxicities and other notes.

Author/Database	Sample	Locoregional Recurrence (LRR)	Overall Survival (OS)	Toxicity	Notes
Haviland JS et al. using the UK Standardisation of Breast Radiotherapy (START) trials of radiotherapy hypofractionation for treatment of early breast cancer (2013) [49] and START Trialists’ Group (2008) [53]	749 women received 50 Gy in 25 fractions of 2.0 Gy; 750 received 41.6 Gy 13 fractions of 3.2 Gy or 3.0 Gy; 737 received 39 Gy in 13 fractions of 3.2 Gy or 3.0 Gy	10-year LRR was 7.4% in the 50 Gy group, 6.3% in the 41.6 Gy group and 8.8% in the 39 Gy group	Not stated	Photographic data and patient surveys at 5-year follow-up suggest reduced side effects after 39 Gy compared to 50 Gy (*p* = 0.01).	Most patients enrolled in these trials received breast-conserving surgery, and a low number of patients received a mastectomy (336 patients in the START A trial (15%) and 177 patients (8%) in the START B trial).
Ko DH et al. using the Christchurch oncology database (2015) [50]	133 patients managed with 40 Gy in 16 fractions	5-year Local recurrence free rate of 97.6%	5-year OS rate of 74.6%	Only one incidence of grade 3 chest wall pain.	Supports using hypofractionation in PMRT.
Poppe MM et al. in a phase 2 prospective trial (2020) [47]	67 patients with stage 2-3A breast cancer who received hypofractionated PMRT with 36.63 Gy in 11 fractions with an optional 4 fractions of 3.33 Gy mastectomy scar boost	5-year LRR was 4.6% as an initial incidence	5-year OS rate of 90%	At 5 years there were no acute or late grade 3- or 4-related toxicities reported for hypofractionated PMRT.	Authors encourage the use of hypofractionation.
Poppe MM et al., RT-CHARM phase 3 randomised controlled trial [48]		nil	nil	nil	Currently investigating outcomes for patients receiving conventionally fractionated and hypofractionated PMRT before or after breast reconstruction.
Tovanabutra C et al. using the Chonburi Cancer Hospital database (2020) [51]	334 patients received hypofractionated PMRT (2.65–2.67 Gy in 15–19 fractions); 128 patients received conventionally fractionated PMRT	5-year LRR was 96.1% for both groups (*p* = 0.993)	5-year OS for the hypofractionated cohort was 64.7%, whilst for conventional therapy it was 71.9%	No differences between groups.	Suggests that hypofractionation could be used in resource-limited settings.
Wang SL et al. in a phase III randomised controlled trial (2019) [52]	820 patients with T3-4 breast cancer or pN2-3 were randomised to either 50 Gy in 25 fractions or 43.5 Gy in 15 fractions PMRT	5-year LRR was 8.3% for hypofractionation and 8.1% for conventional fractionation	Not stated	No differences in acute or late toxicities, except hypofractionated radiotherapy was associated with lower rates of grade 3 acute skin toxicity compared to conventional PMRT (*p* < 0.001).	Breast reconstruction patients were excluded from this trial. Authors suggest hypofractionation provides convenient and effective treatment.
Wong JS et al. in the FABREC study, a prospective randomised trial (2023) [46]	400 patients with stage 0–3 breast cancer were divided into conventionally fractionated PMRT or hypofractionated PMRT (42.56 Gy in 16 fractions)	Not stated	Not stated	At 31.8 months, there were no differences in toxicities between groups, except patients <45 years old were less bothered by side effects (*p* = 0.045) and nausea (*p* = 0.02)	Longer-term results are awaited.

## 5. Strengths and Limitations of This Brief Review

A key asset of this brief review includes that it provides a general overview of contemporary controversial topics within the field of PMRT. Another strength is that we have synthesised a broad range of literature and included studies with large sample sizes and databases in the absence of much randomised data. We have also provided tables/figures which summarise the review’s content, for increased readability and cross-reference. Additionally, we allude to studies currently underway, highlighting the review’s currency, and we outline gaps in the PMRT field that require further research, e.g., genomic profiling scores and the effects of HER 2 receptor status.

This review is limited by being brief and reporting largely retrospective data. It is not a systematic review, meaning that not all databases have been included, so despite all efforts to include key research, we cannot guarantee that we have achieved this. This manuscript did not involve a statistical analysis of included studies, abrogating a quantitative comparison between papers or topics. These limitations may be overcome by conducting a larger, comprehensive meta-analysis or systematic review on existing literature.

## 6. Summary and Conclusions

Radiotherapy may be recommended after a mastectomy for patients with intermediate-risk breast cancer. The aim of such radiation would be to reduce rates of locoregional recurrence and to increase overall survival. Large studies suggest that PMRT is beneficial in intermediate-risk breast cancer [12,13,14,18,20]. Some argue that this benefit is non-existent [11,19] or limited to patients with specific characteristics, including older age [15], or to patients not receiving adjuvant therapy [16]. However, in other studies, PMRT demonstrates benefit alongside adjuvant systemic therapy to be administered in intermediate-risk breast cancer is evident [17,19]. In terms of recommending a timing or type of breast reconstruction best suited to the setting of PMRT, individual patient preferences and circumstances need to be considered alongside a multidisciplinary team. Research into PMRT hypofractionation’s safety and efficacy is ongoing, and its place remains to be fully elucidated, although the retrospective data are very encouraging. Treatment guidelines for PMRT in these settings will be informed by the results of further randomised controlled trials such as SUPREMO, RT-CHARM and FABREC.

## Figures and Tables

**Table 1:** A tabulated summary of the classifications of breast cancer into low, intermediate and high risk. Row 1 is the current St Gallen classification, while the subsequent rows summarise current PMRT guidelines from EviQ, NCCN and ASCO for each risk level of breast cancer. Note that PMRT is also recommended where there are positive surgical margins. * Note that T4 is not explicitly written in the St Gallen’s breast cancer classification but due to the survival of T4 breast cancers in comparison to other cancers in this category, we have grouped it as a high-risk breast cancer. Although T2N0 is classified as an intermediate-risk breast cancer in the St Gallen Classification, it is not in other classifications (see Figure 1).

	Low-Risk Breast Cancer	Intermediate-Risk Breast Cancer	High-Risk Breast Cancer
St Gallen criteria for classifying breast cancer risk [6]	pN0 plus all the below criteria: - T1 - Grade 1 - No vessel invasion - ER and/or PR positive - HER 2-negative - ≥35 years old	pN0 and at least 1 of the following: - Tumour > 2 cm - Grade 2 or grade 3 - Vessel invasion present - HER 2-positive - <35 years old OR pN1 (1–3 positive nodes) and HER 2-negative	pN1 (1-3 positive nodes) and HER 2-positive OR pN2-3 OR * T4
EviQ PMRT guidelines for breast cancer risk [2]	PMRT not recommended	PMRT is recommended in patients with pT3N+. PMRT is controversial but should be “considered” in other patients within this category including pT1-2N1 and pT3N0. T2N0 is not recommended for PMRT.	PMRT is recommended for patients in this category (pT3N+, T4, pN2-3) except for pT1-2N1, which remains controversial, yet “PMRT should be considered”.
NCCN PMRT guidelines for breast cancer risk [3]	PMRT not recommended	PMRT is recommended in patients with pT3N+. PMRT is controversial but should be “strongly considered” in other patients within this category, including pT1-2N1 and pT3N0. T2N0 is not recommended for PMRT.	PMRT indicated for patients in this category (pT3N+, T4, pN2-3) except for pT1-2N1, which remains controversial, yet “PMRT should be strongly considered”.
ASCO PMRT guidelines for breast cancer risk [4,5]	PMRT not recommended.	PMRT is recommended in patients with pT3N+. PMRT is unable to be recommended in pT1-2N1 due to “insufficient evidence”. There is no mention of pT3N0 in these guidelines. T2N0 is not recommended for PMRT.	PMRT is recommended for patients in this category (pT3N+, T4, pN2-3) except for pT1-2N1, which is unable to be recommended due to “insufficient evidence”.

## Data Availability

No new data were created in this study. The original contributions presented in the study are included in the article, further inquiries can be directed to the corresponding authors.
